# Controllable growth of aluminum nanorods using physical vapor deposition

**DOI:** 10.1186/1556-276X-9-400

**Published:** 2014-08-18

**Authors:** Stephen P Stagon, Hanchen Huang

**Affiliations:** 1 Mechanical and Industrial Engineering, Northeastern University, Boston, MA 02115, USA; 2 Mechanical Engineering, University of North Florida, Jacksonville, FL 32224, USA

**Keywords:** Aluminum nanorod, Glancing angle deposition, Oblique angle deposition, Physical vapor deposition

## Abstract

This letter proposes and experimentally demonstrates that oxygen, through action as a surfactant, enables the growth of aluminum nanorods using physical vapor deposition. Based on the mechanism through which oxygen acts, the authors show that the diameter of aluminum nanorods can be controlled from 50 to 500 nm by varying the amount of oxygen present, through modulating the vacuum level, and by varying the substrate temperature. When grown under medium vacuum, the nanorods are in the form of an aluminum metal - aluminum oxide core-shell. The thickness of the oxide shell is ~2 nm as grown and is stable when maintained in ambient for 30 days or annealed in air at 475 K for 1 day. As annealing temperature is increased, the nanorod morphology remains stable while the ratio of oxide shell to metallic core increases, resulting in a fully aluminum oxide nanorod at 1,475 K.

## Background

Metallic nanorods from physical vapor deposition (PVD) have many technological applications, including sensors, through surface-enhanced Raman spectroscopy [1-4], and as an air-tight adhesive for ambient sealing [5]. Due to their unique electrochemical properties, aluminum (Al) nanorods are attractive as electrodes in Li-ion and Al-air batteries [6-8]. Compared to Al powders that are used as the electrodes, Al nanorods grown directly onto current collectors do not require multi-step processing and are better able to accommodate cyclic strain while maintaining current-carrying contact [6,8]. While it is feasible to grow Al nanorods using chemical vapor deposition or template electro-deposition [7,8], PVD can offer better control of purity, alignment, and morphology [6,9]. However, there have been few reports on the growth of Al nanorods using PVD [6,10], without identifying the growth mechanisms and thereby without the control of nanorod diameter.

Before proposing a mechanism to control the diameter of Al nanorods, we must first assess the current state of understanding and determine why the controllable growth of Al nanorods has not been reported so far. Based on modeling studies - including atomistic simulations and theoretical formulations - the growth of metallic nanorods relies on the kinetic stability of multiple-layer surface steps [11,12]. This stability further correlates with the magnitude of diffusion barriers that adatoms experience when moving over multiple-layer surface step [13,14]. According to quantum mechanics calculations, this diffusion barrier is only 0.13 eV for Al [15], compared to 0.40 eV for copper [16], and as a result, the growth of pure Al nanorods has been predicted to be impossible [11]. In contrast to our model prediction, two experimental studies by Au et al. and Khan et al. [6,10] have realized Al nanorods. In reconciling the modeling prediction and the experiments, we note three pieces of knowledge: (1) oxygen (O) atoms may be present at large quantities in the medium to high vacuum levels of the experimental studies [6,10]; (2) O has been used as a surfactant in thin film growth [17,18]; and (3) Al oxide has a much higher melting temperature than Al, and therefore, the adatom diffusion barrier over the surface steps of Al oxide is much larger than the 0.13 eV of Al.

**Figure 1:**
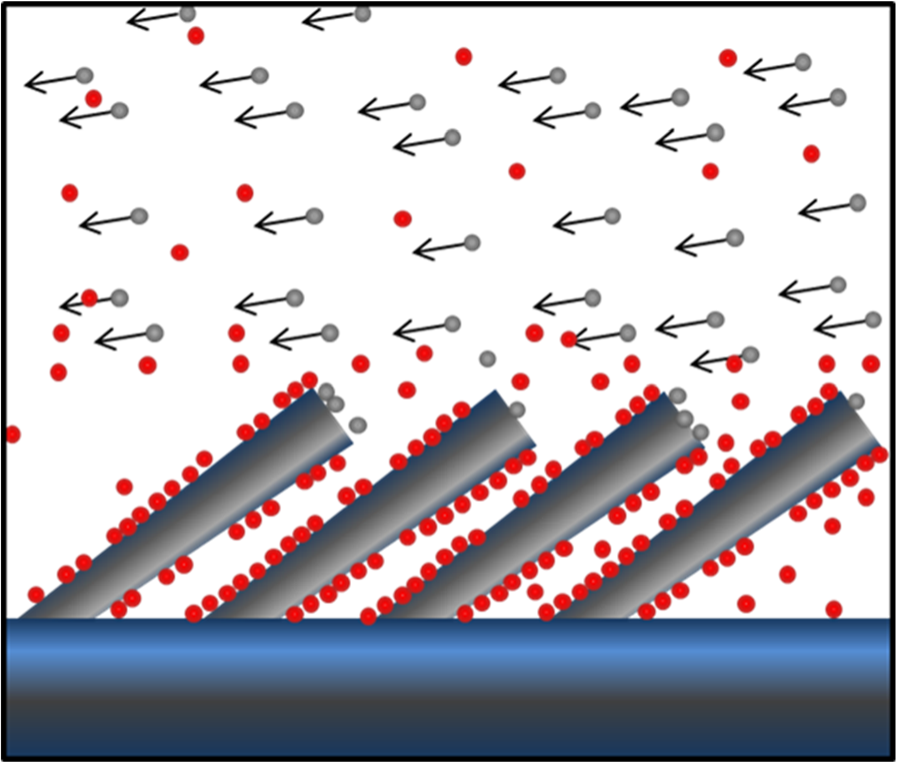
**Oxygen surfactant mechanism schematic.** Schematic of controllably growing Al nanorods (in gray) using physical vapor deposition, with O atoms (red spheres) as surfactant.

In this letter, we first propose the mechanism that enables the growth of Al nanorods using physical vapor deposition based on the three pieces of knowledge noted above. Taking the mechanism to action in combination with existing theory, we go on to grow Al nanorods with controllable diameters through modulation of vacuum levels and substrate temperatures. As schematically shown in Figure 1, our proposal combines the use of glancing angle deposition (GLAD) [19] and the use of O as a surfactant, the amount of which is controlled by the vacuum level.

In the following, we describe how this mechanism functions. Due to the glancing angle incidence, deposited Al atoms land primarily on the top of nanorods or nanorod nuclei (troughs of a rough surface). At low to medium vacuum level, for example 1 × 10 ^-2^ Pa, a large number of O atoms will quickly bind to and decorate the step edges, which are preferential binding sites of surfactant atoms [20]. The stronger local Al-O interactions (relative to Al-Al interactions) will result in a large diffusion barrier for Al adatoms over the surface steps that are decorated by O. Varying the amount of O atoms, through the control of vacuum level, will change either the local chemical composition or the spatial dimension of the Al oxide near the surface steps. Either way, such variation will change the amount of deposited Al atoms that are able to diffuse over the surface steps. Under glancing angle deposition, deposited atoms land primarily on the top of nanorods and their diffusion over the surface steps drives the increase of diameter. As a result, the less diffusion over surface steps, the smaller the nanorod diameter.

## Methods

To demonstrate that the proposed mechanism is feasible, we grow Al nanorods by PVD while varying vacuum levels and substrate temperatures. Our results indeed confirm that the proposed mechanism is feasible, that through its manipulation, Al nanorod diameter is possible, and that Al nanorods grown using this mechanism have the added benefit of thermal stability, which derives from a thin stable oxide shell.

Before presenting the results, we will briefly describe the experimental methods. Al nanorods are grown using electron beam evaporation PVD at varied vacuum levels and varied substrate temperatures. First, Si {100} substrates (Nova Electronic Materials, Flower Mound, TX, USA) are ultrasonically cleaned in acetone, ethanol, and de-ionized water (Millipore, Billerica, MA, USA) and are subsequently placed onto a precision machined mount, for GLAD, at the top of the vacuum chamber. The vacuum chamber is a stainless steel tank that is approximately 40-cm tall and 25 cm in diameter - the source to substrate distance is approximately 30 cm. The source material 99.99% Al (Kurt J. Lesker, Jefferson Hills, PA, USA) is placed in a graphite liner in the electron beam source at the base of the vacuum chamber. For deposition at 1 × 10^-2^ Pa, the high vacuum stage, a turbo-molecular pump, is engaged for only 5 min, after the roughing pressure has been reached; the base pressure reaches 5 × 10 ^-3^ Pa, and the working pressure is 1 × 10^-2^ Pa. The electron beam is then engaged and the deposition rate is monitored and controlled at 1.0 nm/s, via quartz crystal microbalance, to a total nominal thickness of 500 nm. The thickness is measured perpendicular to the source flux, and the measurement represents that of a continuous film. For deposition at 1 × 10^-5^ Pa, the chamber is allowed to remain under high vacuum pumping for 24 h to reach a base pressure of 1 × 10 ^-5^ Pa. To further improve the vacuum, the substrate is blocked from flux via a shutter and chromium (Cr) is deposited onto the chamber walls using the electron beam source. After the deposition of Cr, the base pressure is further improved to 1 × 10^-6^ Pa; the working pressure during deposition is 1 × 10^-5^ Pa. To reach a substrate temperature of 225 K, liquid nitrogen is flowed into the substrate holding fixture and the substrate temperature is measured with K-type thermocouple. The fixture and substrate are allowed to equilibrate to 225 K, and liquid nitrogen is added periodically to maintain the temperature, within a range of 200 to 250 K.

Immediately after the growth is complete the Al nanorods are removed from the deposition chamber and characterized using scanning electron microscopy (SEM) and transmission electron microscopy (TEM). For SEM, Al nanorods are imaged using a FEI Quanta 250 Field Emission Scanning Electron Microscope (FEI, Hillsboro, OR, USA). TEM is performed with Al nanorods that are grown directly onto carbon-coated TEM grids or with Al nanorods drop-coated onto Formvar TEM grids using a FEI Technai operating at 120 KeV.

Thermal annealing experiments are performed in air using a resistance heated tube furnace. The annealing temperature is reached before the samples are placed inside the furnace on an alumina crucible. Timing begins when the sample is placed into the furnace and ends when the sample is removed. TEM samples are annealed while attached to the substrate and are subsequently removed via sonication and drop-coated onto TEM grids.

## Results and discussion

**Figure 2:**
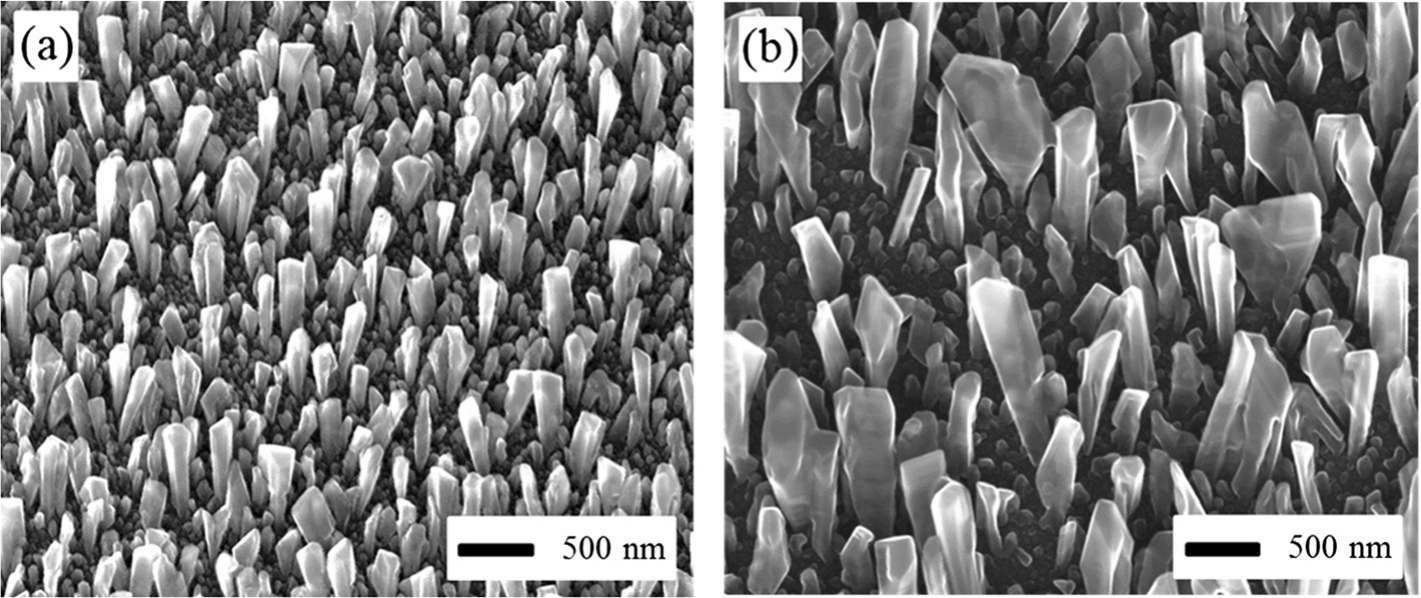
**Dependence of nanorod diameter on vacuum level.** SEM images of Al nanorods grown at **(a)** a low vacuum of 10^-2^ Pa and **(b)** a high vacuum of 10^-5^ Pa; all at a substrate temperature of 300 K.

As the first set of experimental results, Figure 2 contrasts the diameters of Al nanorods grown at different vacuum levels. The only difference in deposition conditions between Figure 2a and Figure 2b is the vacuum level. All other deposition conditions are the same; the substrate temperature is maintained at 300 K, the nominal deposition rate is 1.0 nm/s, and the incidence angle is 86°. Indeed, as we proposed, the lower vacuum leads to a smaller diameter of nanorods, with an average of ~125 nm; the higher vacuum leads to a larger diameter of nanorods - some areas as large as 500 nm. This set of results experimentally demonstrates the feasibility of the mechanism proposed in Figure 1. We recognize that the nitrogen (N) concentration is also high during growth. However, N loses to O in the reaction with Al. Later on, we will also show that indeed, O is present and N is absent in the nanorods, using X-ray energy dispersive spectroscopy (EDS).

**Figure 3:**
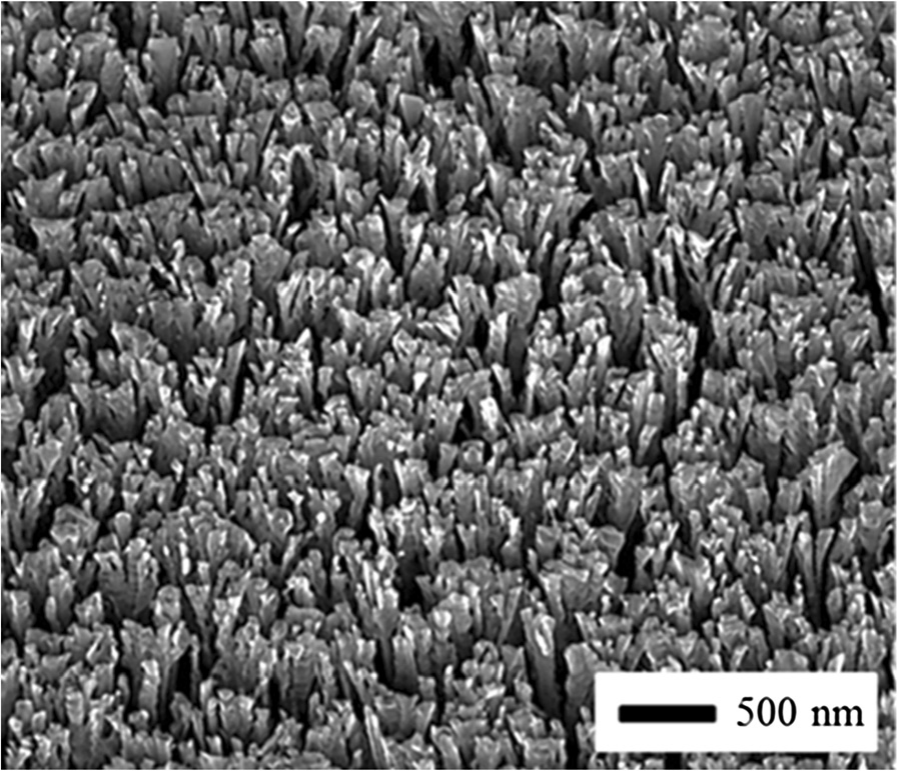
**Low-temperature growth.** SEM image of Al nanorods grown at a low vacuum of 10^-2^ Pa and a low substrate temperature of 225 K.

Motivated by the technological demand for increased specific surface area and nanorods of the smallest diameter [7] and taking the demonstration of controllable growth one step further, we expect that a lower substrate temperature will further decrease the diameter of the nanorods by decreasing the diffusion of adatoms from the tops of nanorods even more than with O alone. As shown in Figure 3 the diameter of Al nanorods is reduced to about 50 nm, which is an order of magnitude smaller than that in Figure 2b. In this case, we note that bunching, or bundling, occurs due to the uncontrolled separation of nanorods [11]; in contrast, the nanorods in Figure 2 are well separated. With the focus on the characteristic diameter, the nanorods that remain separate, or have branched out close to the substrate, are about 50 nm in diameter. We also note that a second cold finger is present in the chamber at a lower temperature than the substrate to mitigate the impingement and condensation of water vapor onto the substrate.

**Figure 4:**
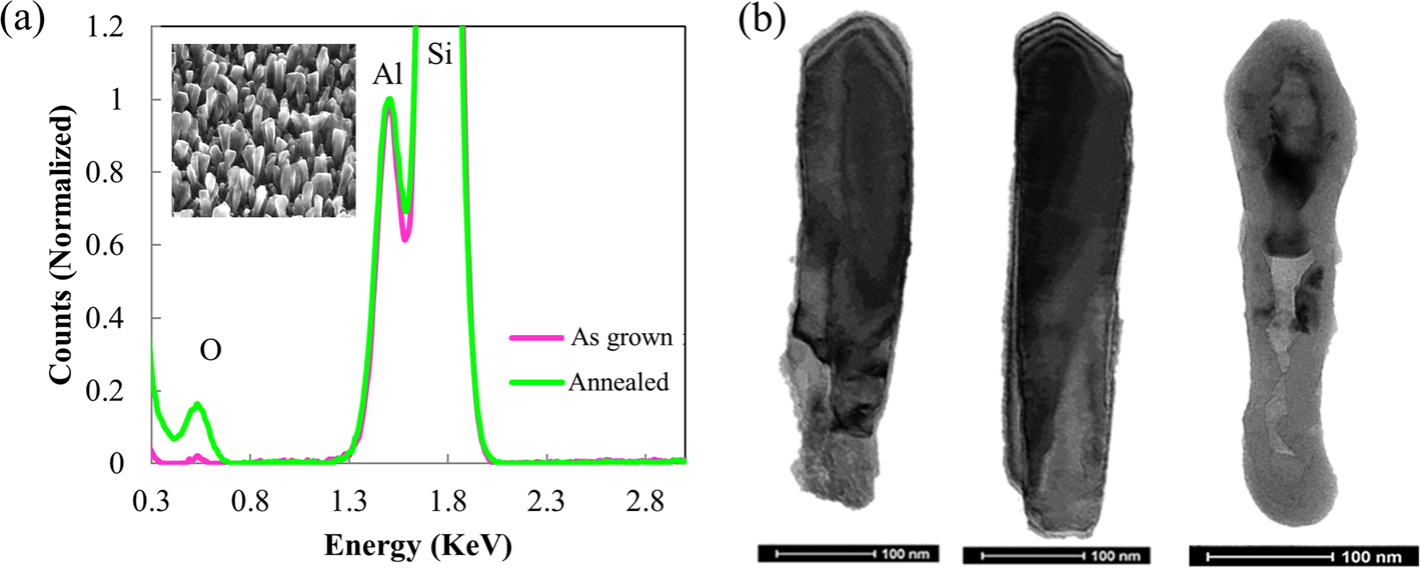
**Analysis of annealed Al nanorods. (a)** EDS spectra of Al nanorods as grown and after annealing at 475 K for 1 day in air, with the SEM image of the annealed Al nanorods as an inset and **(b)** TEM images of Al nanorods before (left) and after the annealing at 475 K (middle) and 875 K (right).

With the use of O as a surfactant, the Al nanorods are likely covered with a layer of Al oxide, which may protect the nanorod morphology from degradation at high temperatures. As the inset of Figure 4a shows, annealing the Al nanorods, which are deposited at room temperature under low vacuum, in air at 475 K for 1 day leads to no visible change in morphology (in comparison to the image in Figure 2a). Our annealing of the same Al nanorods in air at room temperature for 30 days leads to no visible change of morphology, either. The EDS spectra confirm that the nanorods contain Al and O atoms, but no N or other atoms that exist in air or low vacuum. This EDS analysis acts as further evidence to support that O is indeed the dominating chemical element. The accompanying TEM image shows a crystalline core and an amorphous shell of ~2 nm in thickness. Here, the samples are taken immediately from the fabrication chamber to the microscope while under vacuum to prevent oxide formation. Electron diffraction, not shown here, confirms that the core is crystalline aluminum and the shell is amorphous aluminum oxide. Further, TEM images show that the core and shell thicknesses do not change through annealing at 475 K, indicating that the crystalline or amorphous structures remain unchanged (Figure 4b). Pushing the limit of annealing temperature to 875 K (and in air for 30 min), our SEM images do not reveal any visible changes in morphology, but the TEM image in Figure 3b does reveal a marked increase in oxide shell thickness and loss of crystalline core. In passing, we note that annealing at 1,475 K in air for 30 min results in the total conversion of the nanorod into Al_2_O_3_.

In passing, we remark on the impact of the oxide shell. To realize the structures in previous literature studies [6,10], surface oxide formation is necessary. Even with this oxide layer, Al nanorods from PVD perform well in technological applications [6,10]. A level of control of Al nanorod diameter is possible through only substrate temperature control, for the growth of ultra-pure Al nanorods without an oxide shell, but at the expense of extremely low substrate temperatures.

## Conclusions

To summarize, we propose and experimentally demonstrate a mechanism of the controllable growth of Al nanorods using PVD, for the first time, through the use of O as a surfactant. Based on this mechanism, we have achieved the control of Al nanorod diameter from ~50 to 500 nm by varying the amount of O, the vacuum level, and the substrate temperature. The Al nanorods are thermally stable. Their morphology is stable up to 875 K, and their structure (or crystallinity) is stable up to at least 475 K. The controllable growth of thermally stable Al nanorods will enable their applications in technologies such as Al-air and Li-ion batteries and may enable new technologies, such as high-temperature sensing with nanorods, to name just two.

## Competing interests

The authors declare that they have no competing interests.

## Authors' contributions

SPS and HCH designed conceptualized the mechanism and designed the experiments. SPS carried out the fabrication and characterization experiments. SPS and HCH analyzed the results and prepared this manuscript. Both authors read and approved the final manuscript.
